# New Insights of Biological Functions of Natural Polyphenols in Inflammatory Intestinal Diseases

**DOI:** 10.3390/ijms24119581

**Published:** 2023-05-31

**Authors:** Yunchang Zhang, Tianqi Mu, Xiong Deng, Ruiting Guo, Bing Xia, Linshu Jiang, Zhenlong Wu, Ming Liu

**Affiliations:** 1 College of Animal Science and Technology, Beijing University of Agriculture, Beijing 102206, China; zycforward@hotmail.com (Y.Z.);; 2 State Key Laboratory of Animal Nutrition, Department of Animal Nutrition and Feed Science, China Agricultural University, Beijing 100193, Chinabio2046@hotmail.com (Z.W.); 3 Beijing Advanced Innovation Center for Food Nutrition and Human Health, Department of Nutrition and Health, China Agricultural University, Beijing 100193, China

**Keywords:** polyphenols, biological functions, colorectal cancer, enteritis, inflammatory bowel disease

## Abstract

The intestine is critically crucial for nutrient absorption and host defense against exogenous stimuli. Inflammation-related intestinal diseases, including enteritis, inflammatory bowel disease (IBD), and colorectal cancer (CRC), are heavy burdens for human beings due to their high incidence and devastating clinical symptoms. Current studies have confirmed that inflammatory responses, along with oxidative stress and dysbiosis as critical pathogenesis, are involved in most intestinal diseases. Polyphenols are secondary metabolites derived from plants, which possess convincible anti-oxidative and anti-inflammatory properties, as well as regulation of intestinal microbiome, indicating the potential applications in enterocolitis and CRC. Actually, accumulating studies based on the biological functions of polyphenols have been performed to investigate the functional roles and underlying mechanisms over the last few decades. Based on the mounting evidence of literature, the objective of this review is to outline the current research progress regarding the category, biological functions, and metabolism of polyphenols within the intestine, as well as applications for the prevention and treatment of intestinal diseases, which might provide ever-expanding new insights for the utilization of natural polyphenols.

## 1. Introduction

The intestine is the main site for digestion and absorption of dietary nutrients and reabsorption of water and ions, whose homeostasis is extremely crucial for maintaining the health of the host [1]. Meanwhile, the intestine is also the largest immune organ within the human body, composed of multiple immune cells resident in the lamina propria and the gut-associated lymphoid tissue [2,3]. The complicated milieu in the intestinal tract is kept in a subtle balance between pro-inflammation and anti-inflammation to guarantee intestinal homeostasis [4]. Nevertheless, dysfunction of the intestinal mucosal immunity characterized by uncontrolled pro-inflammatory responses and oxidative challenges generally leads to inflammatory intestinal diseases, such as enteritis, inflammatory bowel disease (IBD), and colorectal cancer (CRC) [5,6,7]. 

A series of natural dietary phytochemicals have been found in natural plants, including polyphenols, terpenoids, organo-sulfurs, and phytosterols [8]. Among all these plant-derived biological compounds, polyphenols have been reported to exhibit substantial health-promoting benefits; thus, foods rich in polyphenols are used as functional foods to address cardiovascular and neurodegenerative disease, diabetes mellitus and obesity, as well as osteoporosis through signal cascade regulation properties [9,10]. Likewise, most of the current studies have revealed that the benefits of polyphenols are linked with intestinal health, including anti-oxidative stress, anti-inflammation, and regulation of the intestinal microbiome [11,12]. Thus, functional foods rich in polyphenols are also widely used for the management of inflammation-related intestinal diseases [13,14,15]. There has also been an increasing research interest in the extraction, identification, and purification of natural polyphenols with high bio-efficacy and low side effects for the prevention or treatment of intestinal diseases. Recently, numerous studies focused on the regulation of intestinal health by natural polyphenols have revealed the beneficial effects and relevant mechanisms [11,16,17]. In this scenario, this review critically summarized the categories, metabolism, and biological functions of polyphenols, as well as inflammation-related intestinal diseases, and evaluated the regulatory roles and underlying mechanisms of polyphenols on the onset and process of these diseases, which might provide scientific basis and new insights for the precious use of polyphenols in preclinical and clinical trials.

## 2. Categories and Metabolism of Plant-Derived Polyphenols

The plant-derived compounds consist of a series of bioactive elements, including polyphenols, terpenoids, alkaloids, organo-sulfurs, and phytosterols [18]. Polyphenols, also known as polyphenolic compounds, are one category of the most abundant naturally occurring secondary metabolites derived from phenylalanine or shikimic acid [19]. The structure of polyphenols varies from simple molecules to complex polymers based on the number of enclosed phenolic rings [20]. Meanwhile, polyphenols often occur in conjugated forms with sugars, carboxylic and organic acids, amines, lipids, and even other phenols [21,22]. Thus, more than 9000 polyphenols with different structures and functions have now been identified in the plant kingdom [23]. 

### 2.1. Categories of Polyphenols

According to the different chemical structures characterized by the presence of different numbers of phenolic rings, along with two or more hydroxyl substitutions, these polyphenols customarily can be classified into two major groups, namely flavonoids and non-flavonoids [20]. At least 9000 varieties of flavonoids have been identified [23], all of which share a basic oxygenated heterocycle structure consisting of two aromatic rings bounded by three carbon atoms [9]. In general, flavonoids naturally occur as glycosides, aglycones, or as modified forms, including acetylated, methylated, prenylated, and sulphated derivatives [24,25]. Flavonoids include six subcategories, including anthocyanidin, flavanol, flavanone, flavone, flavanol, and isoflavone [26,27], with cyanidin, epigallocatechin, hesperidin, apigenin, quercetin, and genistein being the most studied represented species, respectively.

Meanwhile, the non-flavonoids are also a large group of polyphenols, which are composed of phenolic acids, stilbenes, tannins, and lignans [26]. Phenolic acids are the main polyphenolic category consisting of benzoic acid, cinnamic acid, and their derivatives [9]. Another important non-flavonoid polyphenol is resveratrol, which belongs to stilbenes and is the research interest due to its novel biological functions [28]. The most common polyphenols with novel biological functions belong to the flavonoids group, such as catechin, epicatechin and derivatives, procyanidins quercetin, kaempferol, as well as genistein. Nevertheless, non-flavonoids polyphenols, such as resveratrol and cinnamic acid, were also reported with functional roles in human health [28,29]. Categories and subcategories of the two large groups were summarized in Table 1.

### 2.2. Bioavailability, Metabolism, and Metabolites of Common Polyphenols

Bioavailability represents the proportion of polyphenols that can be digested, hydrolyzed, and availably absorbed in the gastrointestinal tract [30]. The bioavailability of each polyphenol is different, which is associated with its native chemical structure, extensive modifications, and site of absorption. [31]. Studies carried out in rats and mice showed that anthocyanins and quercetin can be absorbed in the stomach [31,32,33]. Even though the fate of glycosides in the acid milieu is not fully understood yet, one thing that is certain is that most glycosides are probably resistant to acid hydrolysis in the stomach and usually arrive in the intestine [9,34]. In general, aglycones can be absorbed in the small intestine, while most polyphenols exist in the forms of esters, glycosides, or polymers, which cannot be absorbed [31]. Prior to absorption, these polyphenols must undergo transportation by sodium-dependent glucose transporter 1 and hydrolysis by host enzymes in the small intestines [35]. Two host enzymes are required for the hydrolysis of glycosides and release of aglycone, namely the lactase phloridzin hydrolase and cytosolic β-glucosidase, which locates in the brush border of and within the small intestinal epithelial cell, respectively [36]. Except for flavanols, all flavonoids exist in glycosylated forms, which are more absorptive in the small intestines as compared with hydrolyzed aglycone [9,23]. When absorbed and before entering the systemic circulation, polyphenols undergo the settled metabolic detoxication process as many xenobiotics, including methylation, sulfation, and glucuronidation [36]. The circulating polyphenol metabolites are then transported to different organs via systemic circulation and finally excreted in urine [37]. The exception for this is that some of the circulating polyphenol metabolites delivered into the liver further undergo enterohepatic recirculation through the excretion of bile acids [38,39]. 

Even though some polyphenols can be hydrolyzed and absorbed, the bioavailability of polyphenols is quite low in the stomach and small intestines [13,40]. Most of the polyphenols show resistance to acid in the gastrointestinal tract, and very few of them can be hydrolyzed and absorbed in the small intestines [23]. The literature has reported that about 5–10% of polyphenols are absorbed in the small intestine [41], and the remaining polyphenols enter into the colonic lumen and undergo biotransformation by colonic microbiota [42,43]. The intestinal microbiota, equipped with a series of enzymes, can utilize dietary polyphenols in the colon by catalyzing hydrolysis, cleavage, reduction, decarboxylation, demethylation, isomerization, and dihydroxylation reactions [44,45,46]. A fraction of the polyphenols can be degraded into aglycones by the colonic microbiota and is further metabolized into simple aromatic acids, which can be utilized by the host [47,48]. Flavones and flavanones are mainly metabolized into hydroxyphenylpropionic acids, whereas metabolites of flavanols are phenylvalerolactones and hydroxyphenylpropionic acids [48]. Phenylpropionic acids undergo further metabolism to benzoic acids [49]. For example, catechin, epicatechin, epigallocatechin, and EGCG, the most common flavanols, can be metabolized into 3-hydroxyphenylacetic acid, 3-hydroxyphenylpropionic acid, 3,4-dihydroxyphenylacetic acid, and 3-hydroxyphenyl-γ-valerolactone [50]. Both anthocyanidin and quercetin can be metabolized into protocatechuic acid [50]. In addition, daidzein, resveratrol, gallotannin, and ellagitannin can be metabolized into equol, lunularin, gallic acid, and urolithin, respectively [50]. The metabolism of polyphenols also supports the notion of symbiosis between the host and the commensal microbiome [51]. Quite a few of the microbial metabolites of polyphenols are excreted with feces, while the absorbed metabolites share the same fate with metabolized ones in the small intestines. However, whether or not metabolized or modified by the intestinal microbiota increases the bioactivity of polyphenol derivatives still remains controversial [44].

## 3. Inflammation-Related Intestinal Diseases and Corresponding Experimental Models

The intestinal mucosal barrier, consisting of physical, chemical, biological, and immunological barriers, is a complex semipermeable barrier that allows the absorption of nutrients and limits the transport of potentially harmful antigens and microorganisms [52]. Meanwhile, this barrier also plays an essential role in immune sensing, termed intestinal mucosal immunity. Under physiological conditions, the intestinal microbiome and host immune system remain in homeostasis to maintain the intestinal health [53]. However, deficiency of the host immune system or exogenous stimuli, such as pathogen infections, may lead to dysbiosis and dysfunction of the intestinal mucosal immunity, which ultimately results in aberrant inflammatory responses [54]. In such circumstances, inflammation-related intestinal diseases happen, such as enteritis, IBD, and CRC. In consideration of the increasing incidence and prevalence of inflammation-related intestinal diseases and the crucial roles of intestine health, it is quite urgent to explore the underlying pathogenesis of these diseases. Evidence obtained from clinical trials may provide valuable data regarding the onset and process of diseases; nevertheless, so far, this is not capable of identifying the precise mechanism. With the development of current biological technology in animal and cell models, the complexity of the mechanism has gradually been uncovered. Recently, various chemical and infectious animal models of enteritis, IBD, and CRC have been introduced and developed in an effort to provide further insights and therapeutic options against these diseases [55,56].

### 3.1. Clinical Classification of Inflammation-Related Intestinal Diseases

Generally, the most common intestinal disease is IBD, a non-infectious, chronic, and relapsing inflammatory disorder of the gastrointestinal tract with a multifactorial pathophysiology [57]. Two typical phenotypes have been classified regarding the pathogenic sites, namely ulcerative colitis (UC) and Crohn’s disease (CD). Inflammatory responses happen at any segment of the gastrointestinal tract, especially in the terminal ileum and perianal regions of CD patients, while the pathogenic site of UC patients is usually limited in the colon and rectum, with the distal colon and rectum being the most severely affected [58]. Although both innate and adaptive immune systems are involved in CD and UC, the two types of IBD differ in T cell-mediated adaptive immune responses, where T helper 1 (Th1) and Th17 cell responses are active in CD instead of Th1 and Th2 in UC [59]. Nevertheless, CD and UC also share overlapping pathological and clinical symptoms, such as diarrhea, abdominal pain, cramping, rectal bleeding, bloody stool, weight loss, spontaneous remission, and relapsing inflammation [60,61]. In addition, it has been reported that patients with long-term uncured or relapsing IBD show high preference to develop into CRC than general populations, which is considered the fourth most common cancer in the world over the last few decades [62]. 

Another type of inflammation-related intestinal disease is enteritis, wherein inflammatory responses happen in the small intestines as compared with UC and CRC. Typical symptoms of enteritis include abdominal pain, cramping, fever, and diarrhea. Of note, *Clostridium difficile* (*C. difficile*)-induced enteritis is the most commonly observed enteritis with dramatically increasing incidence [63]. Hemorrhagic enterocolitis induced by *Escherichia coli* (*E. coli*) can also be considered enteritis, which causes inflammation and bleeding in the small intestine. This kind of inflammatory intestinal disease is less observed and with low preference to develop into cancer. However, prolonged enterocolitis ultimately results in necrotizing enterocolitis, which frequently happens in ill and premature infants.

### 3.2. Animal Models of Inflammatory Intestinal Diseases

Dextran sodium sulfate (DSS) is a synthetic sulfated polysaccharide with a ranging molecular weight between 5–1400 kDa, which showed high relevance with the severity of colitis along with its duration and dosage [64,65]. DSS-induced colitis is an animal model for human UC, as continuous administration in drinking water induces similar symptoms and Th cell responses [55]. DSS acts as a direct chemical toxin to the colonic epithelial cells, which leads to the breakdown of mucosal integrity, resulting in the exposure of mucosal and submucosal immune cells to luminal antigens and inflammatory responses [66]. Acute, chronic, and relapsing models of UC can be achieved by modifying the concentration and frequency of DSS administration [67]. In addition, DSS-induced colitis spontaneously recovers once termination of administration, which allows for another mouse model for the mechanisms in the recovery phase [58]. Nevertheless, DSS administration is not sufficient to induce colitis-associated cancer, namely CRC, which needs to be utilized in combination with azoxymethane (AOM). AOM can be metabolized into methylazocymethanol (MAM), a highly reactive alkylating species, which will induce DNA mutagenesis once taken up by colonic epithelial cells [68,69]. The injection of AOM and administration of DSS is a short two-step timeline and accurate model, providing both convincing mechanical and pathologic evidence for CRC [56].

Histopathological changes in acetic acid-induced colitis include transmural necrosis, edema, and goblet cell depletion, similar to UC patients [70]. Thus, rectal administration of acetic acid is a well-established animal model as DSS. Physical destruction of colonic mucosal integrity starts within 4 h post acetic acid challenge, and inflammatory responses follow to accelerate mucosal damage via the release of pro-inflammatory cytokines and reactive oxygen species (ROS) [71]. Furthermore, 2,4,6-trinitrobenzenesulfonic acid (TNBS) is a tissue protein-binding hapten in the intestine and can elicit a number of inflammatory responses [55]. Studies have identified that TNBS-type colitis comprises two forms of IBD, as activation of Th1, Th2, and Th17 cell responses were all observed [66]. Even though increased mucosal thickness instead of mucosal damage was observed, which indicated CD-type clinical features of TNBS-induced colitis [66]. Lipopolysaccharide (LPS) is a structural component of the outer membrane of most Gram-negative bacteria. Actually, LPS is a classical ligand for Toll-like receptor 4 (TLR4), activation of which leads to activation of the nuclear factor-κB (NF-κB) signal pathway, followed by nuclear translocation of p65 and subsequent transcriptional activation of downstream inflammatory cytokines [72]. Intraperitoneal injection of LPS induces systemic inflammatory responses termed sepsis [73]. What differs from DSS, acetic acid, and TNBS models is that LPS-induced inflammatory intestinal damage is generally limited within the small intestines instead of the colon [74]. 

Lines of evidence indicate that intestinal bacteria, especially pathogens, play crucial roles in the onset and development of IBD. Epidemiological and clinical studies have observed an elevated abundance of *E. coli* in the intestines of IBD patients and identified enteropathogenic *E. coli* (EPEC) and enterohemorrhagic *E. coli* (EHEC) as the main inducers of CD [75,76]. In addition, *Citrobacter rodentium* (*C. rodentium*), a naturally mouse-restricted enteric pathogen, shares 67% of its genes with EHEC and EPEC, including the locus of enterocyte effacement (LEE) pathogenicity island that encodes the type III secretion system (T3SS) [77,78]. *C. rodentium*, along with EPEC and EHEC, belongs to the attaching and effacing (A/E) pathogen family, whose pathogenesis is achieved by forming the A/E lesions, followed by injecting effector proteins into the host cell via T3SS, ultimately inducing a robust Th1 and Th17 cell responses [79,80]. Thus, oral administration of the abovementioned pathogens establishes excellent animal models for the underlying pathogenesis and potential therapeutic options of human CD. Unlike A/E pathogens, toxigenic *C. difficile* infection usually manifests as pseudomembranous enterocolitis, which frequently occurs after antibiotic use and thus causes antibiotic-associated diarrhea [81]. 

In vivo animal models are of great value for the laboratory investigation of underlying pathogenesis for enteritis, IBD, and CRC. These chemically induced models are frequently used in the current field of enterocolitis due to their reproducible properties and convenience of operating [67]. However, this chemical reagent-induced enterocolitis also showed disadvantages regarding the interaction with host intestinal microbiota when exploring dysbiosis-induced enterocolitis as inflammatory responses come after the epithelium damage. Thus, infectious animal models by non-fatal pathogens, such as *C. difficile*, *E. coli*, and *C. rodentium*, have been utilized in an effort to mimic intestinal dysbiosis and relevant intestinal mucosal damage. In addition to these in vivo animal models, in vitro cell models, such as subculture or isolated epithelial cells and immune cells, are also efficient tools for the direct investigation aiming at intestinal physical and immunological barriers. The animal models for each enterocolitis were summarized in Table 2.

## 4. Therapeutic Applications of Polyphenols in Inflammatory Intestinal Diseases

### 4.1. Potential of Polyphenols in Intervention of Inflammation-Related Intestinal Diseases

Generally, the widely accepted theory is that genetic factors, intestinal dysbiosis, environmental factors, and aberrant inflammatory responses are the main inducible factors in the initiation and/or progression of inflammation-related intestinal diseases [58]. In consideration that environmental factors interact with the intestinal microbiota whose dysbiosis results in the over-activation of inflammatory responses where almost genetic factors are involved [94,95,96], thus dysfunction of intestinal mucosal immunity represented by uncontrolled inflammatory responses contributes mostly to the onset and development of inflammation-related intestinal diseases. Actually, aberrant innate and adaptive inflammatory responses are common consequences in intestinal diseases, mostly represented by the activation of NF-κB and up-regulated levels of pro-inflammatory cytokines, including tumor necrosis factor α (TNF-α), interferon γ (IFN-γ), and interleukins [97]. In addition, it is evident that ROS produced by inflammatory cells are key factors in the progression of inflammatory disorders by serving as signaling molecules and mediators [98], indicating the critical role of oxidative stress in the pathogenesis of intestinal diseases [99]. Elevated ROS levels and imbalanced redox status were convincingly observed in inflammation-related intestinal diseases, while the administration of antioxidants contributes to the remission of these diseases [100]. 

The conclusive signature of biological functions of polyphenols is anti-oxidative stress, achieved through neutralizing free radicals by donating an electron or hydrogen atom from hydroxyl groups [101], reducing highly reactive hydroxyl radicals-induced oxidation by chelating with Fe^2+^ [102], regeneration of essential vitamins [103], and activation of nuclear factor erythroid 2-related factor 2 (Nrf2) related antioxidant system [104]. In addition, numerous sources also reported the anti-inflammatory roles of polyphenols. Mechanically, these polyphenols regulate intestinal inflammatory responses by inactivating NF-κB, modulating mitogen-activated protein Kinase (MAPK), and phosphatidylinositide3-kinases/protein kinase B (PI3K/AkT) signaling cascades, thus reducing synthesis and release of pro-inflammatory cytokines [105]. Furthermore, to function as antioxidants and immunomodulatory regulators, polyphenols must be hydrolyzed and absorbed in the intestines. As previously reported, a large proportion of polyphenols enter into the colon and undergo microbial biotransformation [42]. Meanwhile, polyphenols also interact with intestinal microbiome by modulating its composition and construction, which mainly inhibits the growth of pathogens and promotes the proliferation of *Akkermansia*, *Lactobacillus*, and *Bifidobacterium*, thus serving as prebiotics [106,107,108]. In turn, certain probiotic strains are equipped with tannase, α-L-rhamnosidase, and phenolic acid reductase, which are responsible for the bio-transformation of polyphenols into bioactive phenolic metabolites [109]. In the presence of these enzymes, probiotics can utilize these polyphenols to improve their fitness and persistence in intestinal niches [109]. Based on the abovementioned properties, therapeutic or preventive options using polyphenols targeting intestinal inflammatory responses, oxidative stress, and regulation of intestinal microbiome may be potential efficient intervention approaches against inflammation-related intestinal diseases.

### 4.2. Interventional Options of Polyphenols in Inflammation-Related Intestinal Diseases

Functional foods and their extracts rich in natural polyphenols, such as fruits, coffee, vegetables, and whole grains, have been wildly applied in clinical trials. Anthocyanidins are a group of flavonoids that exist in berries. Anthocyanin-rich bilberry extract was demonstrated to ameliorate disease activity in UC patients [110]. Further study showed anthocyanin-rich bilberry extracts reduced TNF-α and IFN-γ, as well as phosphorylated NF-κB levels, while enhanced levels of IL-22 and IL-10 in colonic biopsies of UC patients [111]. Another clinical study showed that supplementation with anthocyanin-rich purple corn could improve infliximab-mediated disease remission in IBD [112]. One study confirmed that green tea extract enriched in EGCG was an effective supplement for the chemoprevention of relapse of metachronous colorectal adenomas [113]. Resveratrol is a natural polyphone found in grapes, red wine, and berries. A randomized, double-blind, and placebo-controlled pilot study has confirmed that resveratrol capsules treatment increased anti-oxidative capacity, decreased serum malondialdehyde (MDA) level and disease activity, and increased quality of life in patients with UC [114]. Resveratrol was also reported to potentially improve the therapeutic outcomes in patients suffering from CRC when used either alone or as a combination therapy [115]. In addition, clinical trials also reported that daily oral supplementation of resveratrol elicited anti-carcinogenic effects, thus meriting further clinical evaluation as a potential colorectal cancer chemo-preventive agent [116]. EA is a natural polyphone extracted from pomegranate, berries, walnut, and some other nuts. Supplementation with pomegranate juice could decrease the level of fecal calprotectin, a positive linear correlation marker for defining subclinical persistent mucosal inflammation in IBD patients [117]. In addition, oral administration with EA significantly decreased the levels of serum MDA and IL-6 and improved gastrointestinal symptoms in patients with irritable bowel syndrome [118].

More recently, robust experimental studies have been performed to investigate the protective or preventive effects of polyphenols in inflammation-related intestinal diseases based on the persuasive regulatory roles in oxidative stress, inflammation, and dysbiosis. Herein, we selectively summarized some flavonoid and non-flavonoid species in treatment of inflammatory intestinal diseases via in vivo and in vitro laboratory models. The summarized results are listed in Table 3.

Cyanidin-3-Glucoside (C3G) is one of the anthocyanins which can be hydrolyzed into cyanidin (Cy), both of which were reported to improve clinical symptoms and reverse the colonic histological changes in TNBS-challenged mice [119]. In addition, C3G improved DSS-induced body weight loss, colon length shortening, and morphology of colonic mucosa [120]. However, intraperitoneal injection with C3G showed no effects against DSS-induced symptoms except for decreases in pro-inflammatory cytokines and an increase in the regulatory T cell (Treg) population in the colon [121]. In vitro analysis revealed that C3G significantly decreased *TNF-α* and *IL-6* mRNA levels by inactivation of NF-κB in THP-1 [122]. Except for C3G, pelargonidin 3-Glucoside (P3G) also showed beneficial roles in inflammatory intestinal diseases. Oral therapy with P3G reversed DSS-induced diarrhea, bloody stools, erosion of mucosal epithelium, crypt atrophy, loss of villi and goblet cells, as well as inflammatory cell infiltration in the colon of rats [123]. Meanwhile, the beneficial roles of P3G may be associated with anti-oxidative stress, anti-inflammation, and modulation of gut microbiota [124]. Mechanically, pretreatment with P3G inhibited phosphorylated activation of NF-κB and MAPK in LPS-stimulated RAW264.7 cells [125]. P3G-enriched extracts also decreased cell viability via cytostatic arrest of the cell cycle at G1 of SW480 cell and suppressed AOM-induced formation of aberrant crypt foci in the colon, partly via induction of apoptotic caspase 3 [126].

Procyanidin, which is formed from catechin and epicatechin, belongs to proanthocyanin. Several studies have shown the protective effects of procyanidins against DSS-induced murine colitis, which were associated with increased goblet cells, enhanced claudin 1, anti-oxidative enzymes, and short chain fatty acid (SCFA) levels, as well as decreased mRNA levels of pro-inflammatory cytokines [127,128]. Meanwhile, procyanidin treatment activated the AMPK/mTOR/p70S6K signal pathway, thus alleviating DSS-induced colitis by promoting cell proliferation [129]. EGCG is a major bioactive polyphenol in green tea. Several studies revealed the critical roles of EGCG in alleviating DSS-induced clinical manifestations, including intestinal permeability, histopathological changes, and inflammatory cells infiltration in the colon [130], decreasing pro-inflammatory cytokine levels, maintaining Th1/Th2 balance, and inactivating TLR4-NF-κB signaling pathway [131]. Another study indicated that increased abundance of SCFAs-producing microbiota, such as *Akkermansia*, may also be responsible for the beneficial roles [132]. In addition, two studies revealed the therapeutic effects of EGCG in TNBS-induced murine colitis via inhibiting the activation of NF-κB, mast cells and macrophage activation [133,134]. Dietary supplementation with EGCG improved acetic acid-induced colitis, as indicated by colon mucosal damage index and histological scores, and decreased levels of NO, MDA, TNF-α, IFN-γ, p65, as well as increased superoxide dismutase (SOD) activity [135]. EGCG treatment significantly decreased the mean number of aberrant crypt foci and tumor load, as well as increased the abundance of *Bifidobacterium* and *Lactobacillus* in AOM/DSS-induced CRC [136]. In addition, EGCG inhibited genes involved in septum formation, DNA segregation, and cell division in *C. perfringens* in a dose-dependent manner [137]. Consistently, EGCG effectively prevented *C. difficile*-infected mice from death and severe colitis via remodeling the microbial community and inhibiting the transcription of microbial virulence genes (*luxS* and *tcdA*) [138,139]. EGCG also showed protective roles in reducing *E. coli* translocation across IPEC-J2 monolayers by inducing the secretion of porcine B-defensin-1 and -2 and inactivation of the p38 MAPK signal pathway [140].

Oral administration with apigenin alleviated colon length shortening, decreased levels of colonic myeloperoxidase (MPO), alkaline phosphatase (AKP), TNF-α, IL-6, and restored intestinal microbiome in TNBS and DSS colitis models [141,142]. Further studies showed apigenin inhibited the secretion of IL-18 and IL-1β by regulating the canonical and non-canonical NOD-like receptor thermal protein domain associated protein 3 (NLRP3) signal pathways [143]. Apigenin was also demonstrated with potential anti-tumor effects on multiple human cancer cell lines. One study revealed the anti-tumor effects in the progress of CRC were associated with suppressing cell proliferation, migration, and invasion via the Wnt/β-catenin signaling pathway [144]. Except for the commonly used apigenin, tangerine and acacetin also showed protective roles in DSS-induced colitis. Tangerine could enhance the expression of colonic claudin 1 and zonula occluden-1 (ZO-1), increase the abundance of *Lachnospiraceae* and *Lactobacillaceae*, and decrease the abundance of *Enterobacteriaceae* and *Alistipes* [145]. Acacetin could reverse DSS-induced increases in pro-inflammatory cytokines, such as *IL-1β*, *TNF-α*, *IL-6*, and *inducible nitric oxide synthase* (*iNOS*), and decrease microbiota diversity as well [146].

Hesperidin and naringin are natural flavonoid compounds that occur in citrus fruits. Several studies suggested that both hesperidin and naringin could alleviate DSS-induced colitis in mice by improving the integrity of the colon, decreasing the expression of pro-inflammatory cytokines, and elevating the expression of colonic tight junction (TJ) proteins [147,148]. Meanwhile, oral administration of hesperidin and naringin reversed the DSS-disturbed microbial community in the colon and increased the ratio of Firmicutes/Bacteroides [149,150,151,152]. In addition, naringin inhibited the activation of MAPK and NLRP3 inflammasome, as well as endoplasmic reticulum (ER) stress-induced autophagy [147,148]. Orally administered with naringin also prevented AOM/DSS-induced carcinogenesis without significant side effects [148]. Supplementation with hesperidin attenuated LPS-induced inflammatory responses and mucosal damage in the small intestines of broilers, as reported [153].

Kaempferol was widely used in DSS-induced colitis due to its anti-inflammatory property. Treatment with kaempferol alleviated DSS-induced body weight loss, bloody stool, shortened colon, colonic morphological damage, and up-regulated pro-inflammatory cytokines. Mechanically, kaempferol decreased serum LPS concentration, inactivated the downstream TLR4-NF-κB signal pathway, and restored microbial community [154]. The same positive outcomes were also observed in the fecal microbiota from kaempferol-treated mice [154]. Compared to the LPS group, kaempferol dramatically restored transepithelial resistance (TEER), evaluated the expression of TJ proteins, and inactivated of NF-κB signal pathway in Caco2 cells [155]. Kaempferol was also reported to up-regulate expression and inhibit the methylation of DACT2, a tumor suppressor gene, and inactivate the Wnt/β-catenin pathway, thus slowing down and reversing CRC tumorigenesis in an AOM/DSS induced-tumorigenesis model combined with in vitro analyses [156].

Quercetin is another member of flavanol that exists in vegetables and fruits. Supplementation with dihydroquercetin significantly reversed DSS-induced colitis in mice via down-regulating levels of *IL-1β*, *IL-6*, *TNF-α*, and up-regulating serum IL-10, colonic ZO-1, occludin, and *Lactobacillus* levels [157]. The literature also reported that quercetin alleviated DSS-induced murine colitis by increasing the expression of the glutamate-cysteine ligase catalytic subunit (GCLC) and serum glutathione level [158]. Supplementation with quercetin attenuated LPS-induced intestinal injury and decreased pro-inflammatory cytokines and oxidative stress indices. Further analysis revealed that quercetin evaluated the expression of intestinal TJ proteins, inhibited apoptosis of intestinal epithelial cells, and increased the abundance of SCFAs-producing bacteria [159,160]. Quercetin dramatically decreased the number and size of colon tumors in AOM/DSS-induced murine CRC [161]. The AOM rat model revealed that quercetin inactivated the PI3K-Akt signal pathway to reduce proliferation, increase cell apoptosis, and suppress the formation of early preneoplastic lesions in colon carcinogenesis [162]. In addition, one study showed that quercetin improved *C. rodentium*-induced murine colitis by suppressing production of pro-inflammatory cytokines and enhanced the populations of *Bacteroides*, *Bifidobacterium*, *Lactobacillus*, and *Clostridia* [163]. Quercetin was also reported to prevent *E. coli* adhesion to epithelial cells by suppressing focal adhesions and inactivation of NLRP3 in Caco2 cells [164,165]. 

Supplementation with genistein alleviated body weight loss, shortened colon, and inflammation, which skewed M1 macrophages towards M2, and decreased the mRNA levels of pro-inflammatory cytokines, thus attenuating DSS-induced colitis in mice [166]. Another study reported the beneficial effects against DSS-induced colitis were achieved via ubiquitination of NLRP3 inflammasome [167]. Furthermore, the anti-tumor effects were also reported in AOM/DSS-induced CRC in high-fat mice through the PI3K/AKT/FOXO3 or SIRT1/FOXO3 signal cascades [168,169]. Moreover, individual supplementation with genistein or combined with hesperidin in feed was reported to attenuate LPS-induced inflammatory responses and mucosal damage in the small intestines of broilers [153]. The therapeutic effects of genistein in TNBS-induced colitis were also reported, as accompanied by decreased cyclooxygenase-2 (Cox-2) and MPO levels [170].

Oral administration of resveratrol significantly alleviated DSS-induced laboratory symptoms in mice and reduced mRNA levels of pro-inflammatory cytokines, as well as diminished p38 MAPK activation [171]. Another study showed that resveratrol alleviated DSS-induced colitis by down-regulating protein abundance involved in autophagy and up-regulating levels of phosphorylated mTOR and SIRT1 [172]. Intraperitoneal administration of resveratrol to rats also significantly improved TNBS-induced colon injury, decreased MDA level, and increased glutathione peroxidase (GSH-Px) and catalase (CAT) activity [173]. Resveratrol was also reported to alleviate LPS-induced enteritis in broilers and ducks via regulation of Nrf2 and NF-κB signaling pathways [174,175]. An AOM/DSS-induced CRC model indicated the positive outcomes, which were associated with modulating the balance of the colonic microbial community, inhibiting histone deacetylases, and decreasing the populations of Th1 and Th17 cells [176]. Additionally, resveratrol also showed beneficial roles in weaned piglets by down-regulating TLR4, inhibiting the release of *IL-1β* and *TNF-α*, and increasing the secretion of immunoglobulin [177]. As a protein kinases inhibitor, PIC can modify a series of cellular targets and play essential roles in anti-inflammation and anti-tumorigenesis. PIC treatment significantly relieved DSS-induced experimental colitis. Mechanically, the character of PIC is implemented through inhibiting the activation of pro-inflammatory signal mediated by NF-κB and NLRP3 [178], inhibiting while enhancing the expression of pro-apoptotic and TJ proteins, respectively [179,180]. Further analysis showed that PIC treatment modulated the balance of microbiota, increased the abundance of *Akkermansia* and *Lactobacillus*, and decreased the abundance of *Spiroplasmataceae* and *Acholeplasmataceae* [178].

EA has been reported to protect mice from DSS-induced acute and chronic colitis by inactivation of p38 MAPK, NF-κB, and STAT3 signaling, as well as releasing of pro-inflammatory cytokines [181]. Administration of microspheres of EA improved morphology of colonic mucosa by alleviating oxidative stress in DSS-induced colitis in rats, particularly in a dose-dependent manner [182]. Meanwhile, in an intra-colonic administration of TNBS-induced CD model, EA also demonstrated beneficial roles in diminishing the severity and extension of intestinal injuries, increasing mucus production, decreasing neutrophil infiltration and expression of Cox-2 and iNOS, as well as inactivation of p38, JNK, ERK, and p65 [183]. The beneficial effects of EA-enriched pomegranate extract were investigated in TNBS-induced murine colitis, which showed drastically decreased levels of Cox-2, iNOS, and phosphorylation of MAPK and NF-κB [184]. Two more evidence for the application of EA against CD were also reported. EA-enriched pomegranate peel extract was reported to reduce colonic damage and bacterial translocation and promote colonization resistance of the host in *C. rodentium*-induced infectious colitis [185,186]. The protective effects of EA against enteritis have also been investigated. Dietary supplementation of EA regulated the occurrence of intestinal inflammatory responses and increased probiotics abundance, thereby attenuating enteritis in weaned piglets [187]. Fecal microbiota transplantation of an EA-contained diet also showed attenuated intestinal damage and oxidative stress [188]. Meanwhile, as recently reported, EA ameliorated *Clostridium perfringens* (*C. perfringens*)-induced subclinical necrotic enteritis in broilers by inhibiting inflammation and restoring of cecal microbiota, providing further evidence for treatment of human necrotic enteritis [189]. Anti-cancer effects of EA were also explored. An in vitro study based on transcriptome microarray assay showed that EA efficiently inhibited the proliferation of HCT-116 cells by regulating genes involved in cell proliferation, cell cycle, apoptosis, and angiogenesis [190]. Another study also reported the anti-proliferative property of EA in Caco2 cells, which may result from the pro-apoptotic properties via the cytochrome c-dependent mitochondrial intrinsic pathway [191]. Furthermore, EA enhanced the production of ROS and apoptosis, as well as decreased HCT-15 cell proliferation [192]. 

CA is the major hydroxycinnamic acid in coffee. Pre-administration with a CA-enriched diet is beneficial in DSS-induced colitis, which was associated with alleviated inflammatory responses, infiltration of immune cells, activation of Nrf2-dependent antioxidative system, and increased abundance of *Akkermansia* [193,194]. Cellular investigation revealed that CA exerted direct suppressive effects on the activation of bone marrow-derived macrophages (BMDMs) upon exposure to TLRs agonists in vitro [194]. In addition, CA improved colonic mucosal barrier function by enhancing the production of SCFAs and expression of TJ proteins and decreasing the abundance of *E. coli* in a weaning stress-induced porcine colonic inflammatory model [195]. However, except for the abovementioned protective roles of caffeic acid in DSS-induced colitis [196,197], no study has reported the beneficial roles of CA in TNBS- and acetic acid-induced, as well as *E. coli* and *C. rodentium*, infectious colitis models. Otherwise, treatment with CA inhibited the proliferation of HCT-15 cells by arresting cell cycle at sub-G1 phase and induced ROS-relevant cell apoptosis in a dose- and time-dependent manner [198]. Furthermore, in vivo and in vitro analyses revealed that CA effectively suppressed self-renewal capacity, stem-like characteristics, and migratory capacity of colorectal cancer stem cells, which may be relevant to the PI3K-AKT signal [199]. 

CHA is an ester form of CA and quinic acid. Studies using DSS-induced colitis have revealed the protective effects of CHA, all of which were associated with the inactivation of inflammatory signals, such as ERK, JNK, AKT, and STAT3, reduction in pro-inflammatory cytokines, infiltration of immune cells, and apoptosis of colonic epithelial cells [200,201,202]. A TNBS-induced murine colitis model identified that intrarectal injection was more efficient than oral administration. CHA exhibits anti-inflammatory properties through reduction of neutrophil infiltration and inhibition of NF-κB signal, demonstrating the valuable supplement in the treatment of IBD [203]. As with EA, CHA was also reported to alleviate weaning stress-induced enteritis by enhancing the anti-oxidant system and increasing while decreasing the abundance of *Lactobacillus* and *E coli*, respectively [204]. In addition, CHA also inhibited structural damage and inflammatory cytokines and improved antioxidant capacity in the small intestines, thus providing protective effects against *C. perfringens*-induced enteritis [205]. Consistently, CHA treatment decreased levels of *IFN-γ* and *TNF-α* and increased the expression of occludin in jejunum and colon, thus decreasing intestinal permeability in LPS-stimulated rats [206]. More importantly, CHA also served as an antibiotic alternative to kill *E. coli* by directly targeting bacterial cell walls and membrane [207] or inducing apoptosis in *E. coli* via depletion of intracellular ROS [208], even though nearly no studies have been conducted regarding the protective roles of CHA against *E*. *coli*- or *C. rodentium*-induced enterocolitis. In contrast to this, CHA was found to induce the accumulation of ROS in HT-29 and SW480 cells, thus playing a critical role in CRC [209]. Meanwhile, CHA, along with its microbial metabolites including CA, 3-phenylpropionic acid, and benzoic acid, was reported to exert anti-proliferative effects via cell cycle arrest at the S-phase and induce apoptosis via caspase 3 manner in Caco2 cells [210].

**Table 3:** Applications polyphenols in inflammatory intestinal diseases.

Polyphenols	Models	Dosage	Outcomes and Mechanisms	References
C3G	DSS/Mouse	12.5, 25, 50 mg/kg, 1 μg	Improve body weight loss, colon length shortening, and colonic mucosal damage; decrease pro-inflammatory cytokines; increase Tregs population	[120,121]
	TNBS/Mouse	24.2, 48.4, 96.8 mg/kg	Improve clinical symptoms and colonic histological damage	[119]
	THP-1	0.005, 0.05, 0.5, 10 μM	Decrease *TNF-α* and *IL-6* mRNA levels by inactivation of NF-κB signal	[122]
P3G	DSS/Rat, mouse	5, 200 mg/kg	Attenuate diarrhea, erosion of mucosal epithelium, crypt atrophy, and loss of villi and goblet cells; regulation of oxidative stress, inflammatory responses, and intestinal microbiome	[123,124]
	TNBS/Mouse	5 mg/kg	Decrease TNF-α, IFN-γ, IL-6 levels; promote expansion of Tregs and M2 macrophages	[211]
	AOM/Mouse	10–30%	Suppress formation of aberrant crypt foci; induction of caspase 3	[126]
	RAW264.7	10 μM	Inactivation of NF-κB and MAPK	[125]
	SW480	0–40 μM	Decrease cell number via cytostatic arrest of cell cycle at G1-phase	[126]
Procyanidins	DSS/Mouse	5, 10, 125, 200, 250, 500 mg/kg	Increase goblet cells, claudin 1, anti-oxidative enzymes, and SCFAs; decrease levels of pro-inflammatory cytokines; activate AMPK/mTOR/p70S6K signal	[127,128,129]
EGCG	DSS/Rat, mouse	20, 50 mg/kg	Improve intestinal permeability and histopathological changes; decrease immune responses; maintain Th1/Th2 balance; increase abundance of SCFAs-producing microbes	[130,131,132]
	TNBS/Mouse	10, 30 mg/kg	Inactivation of mast cells and macrophage, NF-κB	[133,134]
	Acetic acid/Rat	50 mg/kg	Improve colon mucosal damage index and histological scores; decrease levels of NO, MDA, TNF-α, IFN-γ, and p65; increase SOD level	[135]
	AOM/DSS/Mouse	1%	Decrease number of aberrant crypt foci and tumors; increase abundance of *Bifidobacterium* and *Lactobacillus*	[136]
	*C. perfringens*	100, 250 mg/L	Inhibit genes involved in septum formation, DNA segregation, and cell division	[137]
	*C. difficile*/Mouse	0, 5, 10, 25, 100 μg/mL, 25, 50 mg/kg	Prevent death and severe colitis via remodeling microbial community; inhibit transcription of microbial virulence genes	[138,139]
	*E. coli*/IPEC-J2	50 μM	Reduce *E. coli* translocation through inducing secretion of porcine β-defensin1 and 2; inactivation of p38 MAPK	[140]
Apigenin	DSS/Rat, mouse	3, 150, 250 mg/kg	Relieve intestinal pathological injury; increase goblet cells and mucin secretion; regulate immune responses, increase TJ protein and SCFAs levels; remodel gut microbiota	[141,142]
	TNBS/Rat	1, 3, 10 mg/kg	Amelioration of morphological signs and biochemical markers	[141]
	THP-1	10 μM	Inactivation of NLRP3 inflammasome	[143]
	SW480, HCT-15	0–80 μM	Inhibition of cell proliferation, migration, and invasion via Wnt/β-catenin signal pathway	[144]
Tangerine	DSS/Mouse	0.04, 0.08%	Enhance expression of colonic claudin 1 and ZO-1; regulation of intestinal microbiome	[145]
Acacetin	DSS/Mouse	50, 150 mg/kg	Decrease pro-inflammatory cytokines; increase microbial diversity	[146]
Hesperidin	DSS/Mouse	1, 10, 40, 80 mg/kg	Decrease MPO, MDA, and serum IL-6 levels; reverse disturbed microbial community	[151,152]
	LPS/Broiler	20 mg/kg	Attenuate inflammatory responses and mucosal damage in the small intestines	[153]
Naringin	DSS/Mouse	100 mg/kg	Increase expression of TJ proteins; reverse disturbed microbial community; suppress activation of NF-κB, MAPK, and NLRP3	[147,149,150]
	AOM/DSS	50, 100 mg/kg	Inhibit myeloid-derived suppressor cells, pro-inflammatory cytokines, NF-κB/IL-6/STAT3 signal cascades, and ER stress-induced autophagy	[147]
Quercetin	DSS/Mouse	3 g/kg, 100, 1000, 1500 ppm	Down-regulate levels of *IL-1β*, *IL-6*, and *TNF-α*; up-regulate levels of serum IL-10, colonic ZO-1, occludin, and *Lactobacillus*; increase GCLC and serum glutathione levels	[157,158]
	LPS/Broiler	0.4, 200 mg/kg	Improve intestinal morphology; decrease pro-inflammatory cytokines and oxidative stress indices	[159,160]
	AOM/DSS/Mouse, Rat	30 mg/kg, 4.5 g/kg	Decrease number and size of colon tumors and oxidative stress markers	[161]
	*C. rodentium*/Mouse	30 mg/kg	Suppress production of pro-inflammatory cytokines; enhance populations of *Bacteroides*, *Bifidobacterium*, *Lactobacillus*, and *Clostridia*	[163]
	*E. coli*/Caco2	200 μM	Suppress focal adhesions and inactivation of NLRP3 in Caco2	[164,165]
Kaempferol	DSS/Mouse	50 mg/kg	Attenuate laboratory symptoms; decrease pro-inflammatory cytokines and serum LPS concentration; inactivate TLR4-NF-κB signal; restore microbial community	[154]
	Caco2/LPS	80 μM	Restore TEER; evaluate expression of TJ proteins; inactivation of NF-κB	[155]
	HCT-116, HT29, YB5, SW48	1.25–150 μM	Up-regulate expression and inhibit methylation of DACT2	[156]
	AOM/DSS/Mouse	150 mg/kg	DACT2 epigenetic restoration; inactivate Wnt/β-catenin pathway	[156]
Genistein	DSS/Mouse	5, 10, 15, 45 mg/kg	Attenuate body weight loss and shortened colon; skew M1 macrophages towards M2; decrease pro-inflammatory cytokine levels; ubiquitination of NLRP3 inflammasome	[166,167]
	AOM/DSS/Mouse	50, 150, 450 mg/kg	Inhibition of PI3K/AKT/FOXO3 or SIRT1/FOXO3 signal cascades	[168,169]
	LPS/Broiler	5 mg/kg	Attenuate inflammatory responses and mucosal damage in small intestines	[153]
	TNBS/Rat	100 mg/kg	Decreased Cox-2 and MPO levels	[170]
Resveratrol	DSS/Mouse	20, 80 mg/kg	Alleviate body weight loss, diarrhea, rectal bleeding; decrease mRNA levels of TNF-α and IFN-γ; inactivate p38 MAPK; down-regulate protein abundance in autophagy; up-regulate p-mTOR and SIRT1	[171,172]
	TBNS/Rat	10 mg/kg	Improve colon injury; decrease MDA level; increase GSH-Px and CAT activity	[173]
	AOM/DSS/Mouse	100 mg/kg	Modulate the balance of colonic microbial community; inhibit histone deacetylases; decrease populations of Th1 and Th17 cells	[176]
	LPS/Broiler, duck	400 mg/kg	Alleviate enteritis; increase antioxidant capacity; modulate intestinal immunity; regulate Nrf2 and NF-κB signaling pathways	[174,175]
	*E. coli*/Pig	300 mg/kg	Inhibit release of *IL-1β* and *TNF-α*; increase secretion of immunoglobulin	[177]
PIC	DSS/Rat, mouse	1, 2.5, 5, 10 mg/kg	Inactivation of NF-κB and NLRP3, expression of pro-apoptotic proteins; enhanced expression of TJ proteins; regulate intestinal microbiome	[178,179,180]
EA	DSS/Rat, mouse	2%, 10 mg/kg	Improve morphology of colonic mucosa; inactivation of p38 MAPK, NF-κB, and STAT3 signaling; down-regulation of pro-inflammatory cytokines; alleviate oxidative stress	[181,182]
	TNBS/Rat	10–20 mg/kg	Diminish severity and extension of intestinal injuries; increase mucus production; decrease neutrophil infiltration and expression of Cox-2 and iNOS; inactivation of p38, JNK, ERK, and p65	[183,184]
	*C. rodentium*/Mouse	/	Reduce colonic damage and bacterial translocation; promote colonization resistance	[185,186]
	Weaning/Pig	500 g/t	Regulation of occurrence of intestinal inflammatory responses; promote probiotics abundance	[187]
	*C. perfringens*/Broiler	500 mg/kg	Inhibit inflammation and restore cecal microbiota	[189]
	HCT-116	100 μM	Regulate genes involved in proliferation, cell cycle, apoptosis, and angiogenesis	[190]
	Caco2	1–30 μM	Promote cytochrome c-dependent mitochondrial apoptosis	[191]
	HCT-5	20–120 μM	Enhance production of ROS and apoptosis	[192]
CA	DSS/Mouse	50, 251 mg/kg	Alleviate inflammatory responses; infiltration of immune cells; activation of Nrf2-dependent antioxidative system; increase abundance of *Akkermansia*; inactivation of BMDMs	[193,194]
	Weaning/Pig	250, 500 mg/kg	Improve colonic mucosal barrier function by enhancing production of SCFAs and expression of TJ proteins; decrease abundance of *E. coli*	[195]
	HCT-15	0–2500 μM	Cell cycle arrest at sub-G1 phase; induce ROS-relevant cell apoptosis	[198]
CHA	DSS/Mouse	1 mM, 200 mg/kg	Inactivation of ERK, JNK, AKT, and STAT3; reduce pro-inflammatory cytokines, infiltration of immune cells, and apoptosis of colonic epithelial cells	[200,201,202]
	TNBS/Mouse	20 mg/kg	Reduction in neutrophil infiltration; inhibition of NF-κB signal pathway	[203]
	Weaning/Pig	250, 500, 1000 mg/kg	Enhance anti-oxidant system and TJ protein; increase *Lactobacillus*; decrease *E. coli*	[204]
	*C. perfringens*/Broiler	500 mg/kg	Inhibition of structural damage and release of inflammatory cytokines; improved antioxidant capacity	[205]
	HT-29, SW480	250, 500, 1000, 2000 µM	Induce accumulation of ROS; regulation of Wnt/β-catenin	[209]
	Caco2	50–1000 µM	Anti-proliferative effects via cell cycle arrest at the S-phase; apoptosis via caspase 3 manner	[210]

## 5. Conclusions and Future Directions

The most commonly diagnosed inflammation-related intestinal diseases are enteritis, colitis, and CRC. The three kinds of intestinal diseases have brought great suffering to human beings due to their high incidence, long duration, devastating clinical symptoms, and low curability. In this scenario, numerous intervention strategies using nutritional and non-nutritional biological substances have been performed to investigate efficient approaches, among which polyphenols attract more spotlights owing to their anti-oxidative and anti-inflammatory properties. Indeed, most polyphenol species showed positive outcomes in laboratory investigations and clinical trials. Nevertheless, the current review gave a limited glimpse of some of the represented well-known polyphenols and their applications in inflammation relate intestinal diseases. Furthermore, safety and potential toxicity must be taken into consideration, as notably negative effects were also observed. Thus, extensive studies are still required to elucidate the safe dose of the corresponding polyphenol. Meanwhile, in consideration of the low bioavailability of polyphenols due to the complicated structures and high molecular weight, modifications to improve bioavailability through microencapsulation, nano delivery systems, and microemulsions may be feasible directions. 

## Figures and Tables

**Table 1:** Categories and subcategories of natural polyphenols.

Category	Subcategories	Species
Flavonoids	Anthocyanidin	Cyanidin, Delphinidin, Pelargonidin, Malvidin
	Flavanol	Catechin, Epicatechin, Epigallocatechin, Epigallocatechin-3-gallate (EGCG)
	Flavanone	Naringin, Hesperidin, Taxifolin
	Flavone	Apigenin, Luteolin, Acacetin, Baicalein, Chrysin, Tangeritin
	Flavanol	Kaempferol, Galangin, Morin, Myricetin, Quercetin, Isorhamnetin
	Isoflavone	Biochanin, Genistein, Daidzein, Glycitein
Phenolic acids	Benzoic acid	Ellagic acid (EA), Gallic acid, Hydroxybenzoic acid, Vanillic acid, Protocatechuic acid, Syringic acid
	Cinnamic acid	Chlorogenic acid (CHA), Ferulic acid, Caffeic acid (CA)
Stilbenes	/	Resveratrol, Pterostilbene, Piceatannol (PIC)
Tannins	Condensed tannins	Proanthocyanin
	Hydrolyzable tannins	Gallotannin, Ellagitannin
Lignans	Arylnaphthalene	/
	Aryltetralin	/
	Dibenzylbutane	/
	Dibenzylbutyrolactone	/
	Tetrahydrofuran	/
	Furofuran	/

**Table 2:** Animal models for inflammatory intestinal diseases.

Clinical Type	Inducer	Animal Species	References
UC	DSS	Mouse, rat, rabbit	[82,83,84]
	Acetic acid	Mouse, rat	[85,86]
CD	TNBS	Mouse, rat	[87]
	*E. coli*	Mouse, pig	[88,89]
	*C. rodentium*	Mouse	[79]
CRC	DSS + AOM	Mouse	[56]
Enteritis	LPS	Mouse, pig, broiler	[90,91,92]
	*C. difficile*	Hamster, guinea pig, rabbit, rat, mouse, broiler	[81,93]

## Data Availability

Data are available upon reasonable request.
